# AN ONLINE VIGNETTE STUDY TO EXAMINE THE OUTCOMES OF A PRECLINICAL ALZHEIMER’S DISEASE DIAGNOSIS

**DOI:** 10.1093/geroni/igad104.1567

**Published:** 2023-12-21

**Authors:** Matthew Picchiello, Matthew Wynn, Brian Carpenter

**Affiliations:** Washington University in St. Louis, St. Louis, Missouri, United States; University of California: San Francisco Memory & Aging Center, San Francisco, California, United States; Washington University in St. Louis, St. Louis, Missouri, United States

## Abstract

Preclinical Alzheimer disease (AD) is characterized by abnormal levels of amyloid beta and tau proteins in the brain of a cognitively normal person. There are potential risks and benefits of communicating biological marker (biomarker) risk information for AD using the preclinical AD label. The current study used a vignette methodology to evaluate older adults’ reaction to their hypothetical risk for developing AD. Community dwelling cognitively intact older adults (n = 300, M age = 64.1) completed an online survey, randomized to disclose biomarker results and risk information (with or without the preclinical disease label) pertaining either to heart disease or AD. Participants reported on their perceptions of risk and behavioral intentions based on the Health Belief Model. A series of ANCOVAs and moderated mediation models support the idea that, across disease conditions, a preclinical label does not influence perception of the disease or behavioral intentions. Both groups indicated a similar and proactive intention to engage in more health-promoting behaviors and plan for the future. Results also highlight differences in individual perceptions of AD versus heart disease such that participants in the AD condition perceived their risk information as implying a more severe condition, perceived fewer benefits to knowing their risk, and reported lower self-efficacy about doing anything to address that risk. Despite these perceptions, older adults who received risk information for AD maintained interest in undertaking behavioral changes that may improve their quality of life. These findings have implications for the development of empirically supported disclosure processes for preclinical AD.

